# Chemotherapy Use, Healthcare Resource Utilisation, and Direct Costs in High-Risk Early-Stage ER+/HER2– Breast Cancer in Italy: A Real-World Study Linking Administrative and Pathology Data

**DOI:** 10.3390/cancers18213467

**Published:** 2026-10-27

**Authors:** Carmen Criscitiello, Valentina Perrone, Luca Degli Esposti, Stefania Mazzoni, Kaushal Desai, Kim M. Hirshfield, Jagadeswara Rao Earla

**Affiliations:** 1 Humanitas Research Hospital, 20072 Milan, Italy; carmencriscitiello@hotmail.com; 2 Division of Early Drug Development, European Institute of Oncology, IRCCS, 20141 Milan, Italy; 3 CliCon S.r.l. Società Benefit, Health, Economics & Outcomes Research, 40137 Bologna, Italy; luca.degliesposti@clicon.it (L.D.E.); stefania.mazzoni@clicon.it (S.M.); 4 Merck & Co., Inc., Rahway, NJ 00765, USA; kaushal.desai@merck.com (K.D.); kim.hirshfield@merck.com (K.M.H.)

**Keywords:** administrative databases, chemotherapy, direct costs, early-stage breast cancer, ER-positive, HER2-negative, healthcare resource utilisation, oncology outcomes, real-world evidence, recurrence

## Abstract

**Background/Objectives**: Patients with high-risk early-stage oestrogen receptor-positive/human epidermal growth factor receptor 2-negative early breast cancer (ER+/HER2– eBC) remain at high risk of recurrence despite standard endocrine therapy. Given the limited real-world data in Italy, this analysis evaluated treatments, economic burden and clinical outcomes in routine practice. **Methods**: Administrative healthcare databases linked with pathology data were used to identify patients with ER+/HER2– eBC undergoing surgery. The overall population (Cohort #1) comprised patients identified between January 2010 and June 2024. A subset meeting high-risk criteria constituted Cohort #2 (Grade 3 tumours AND clinical stage T1c–T2/N1–N2 or T3–T4/N0–N2; when TNM components were unavailable, clinical stage II–III patients were included). Healthcare resource utilisation and direct costs were assessed during neoadjuvant, adjuvant, and follow-up phases. Recurrence rate, real-world event-free survival and overall survival were evaluated during the 4 years post-surgery using Kaplan–Meier curves. **Results**: Among 17,731 patients identified as ER+/HER2– eBC (Cohort #1), 366 were classified as high-risk (Cohort #2). The descriptive analysis documented substantial healthcare resource utilisation and direct costs in the high-risk subgroup during both neoadjuvant and adjuvant phases, together with a considerable recurrence burden and reduced survival probabilities. **Conclusions**: This descriptive real-world analysis provides estimates of the clinical and economic burden of ER+/HER2– eBC in the real Italian practice, with a focus on the high-risk subgroup. Post-surgery cost estimates reflect healthcare expenditure during recurrence-free follow-up and do not capture the costs of recurrent or metastatic disease. These findings highlight the healthcare resources required for the management of high-risk eBC and the need for continued efforts to improve clinical outcomes.

## Simple Summary

Patients with high-risk early-stage oestrogen receptor-positive/human epidermal growth factor receptor 2-negative (ER+/HER2–) breast cancer remain at significant risk of recurrence despite standard endocrine therapy. Chemotherapy is recommended in this setting, but limited information is available on how these patients are treated in routine clinical practice in Italy and the related healthcare costs. This real-world study analysed Italian administrative and pathology data to evaluate treatment patterns, healthcare resource utilisation, and direct costs in this setting. The analysis provided descriptive estimates for the overall ER+/HER2– eBC population and its high-risk subgroup. Patients meeting high risk criteria showed a substantial economic burden, along with a considerable risk of recurrence and worse clinical outcomes. Post-surgery costs were assessed during recurrence-free follow-up and therefore did not include expenditures following recurrence or death. These findings highlight the clinical challenges and healthcare resource requirements associated with the management of high-risk early breast cancer, supporting the need for improved therapeutic strategies and further research into their clinical and economic impact.

## 1. Introduction

Breast cancer (BC) remains the most frequently diagnosed cancer in women worldwide and a leading cause of cancer-related mortality [1,2]. In Italy, BC represents the most common malignancy among women, with approximately 55,000 new cases annually, accounting for about 30% of all female cancers, and a lifetime risk of around one in eight women. Despite the high incidence, mortality has progressively declined, with 5-year survival rates exceeding 85–87%, mainly due to screening programmes and therapeutic advances [3,4]. Among BC subtypes, oestrogen receptor-positive/human epidermal growth factor receptor 2-negative (ER+/HER2–) tumours represent the largest proportion of incident cases and account for a substantial share of BC-related morbidity and long-term follow-up needs [5]. In early-stage disease, outcomes are generally favourable with locoregional treatment and adjuvant endocrine therapy (ET) [6,7]. However, ER+/HER2– early BC (eBC) is clinically heterogeneous, with considerable variability in clinicopathological characteristics and recurrence risk [8]. Within this heterogeneous population, a subset of patients presents with adverse clinicopathological features, including nodal involvement, larger tumour size, higher grade, and high proliferative activity, which are associated with an increased risk of disease recurrence. These patients remain particularly challenging to manage because of the persistent risk of recurrence, including late recurrences occurring many years after initial diagnosis [9,10].

Optimising adjuvant systemic therapy in ER+/HER2– eBC requires careful risk stratification and balancing the expected absolute benefit of chemotherapy (CHT) against acute and long-term toxicities. Tumour size and axillary lymph node status are among the strongest predictors of BC outcome, with rising risk as tumour burden increases [11,12]. In endocrine-responsive disease, adjuvant ET remains foundational. Meta-analytic evidence demonstrates that tamoxifen and aromatase inhibitors reduce recurrence and BC mortality, yet residual risk persists for many patients, particularly those with high-risk features [13,14,15]. More recently, persistent residual recurrence risk despite ET has led to the development of adjuvant CDK4/6i-based strategies. Abemaciclib plus ET improved invasive disease-free survival and overall survival in node-positive, high-risk HR+/HER2– eBC in the monarchE trial [16,17]. Ribociclib plus a nonsteroidal aromatase inhibitor was associated with better invasive disease-free survival in stage II–III HR+/HER2– eBC in the NATALEE trial [18], with the absolute benefit increasing at 5 years and a clinically relevant overall survival benefit emerging at 6 years of follow-up [19,20]. In parallel, giredestrant has emerged as a promising oral selective oestrogen receptor degrader under clinical development, although it has not yet received regulatory approval. In the phase III lidERA trial, giredestrant reduced the risk of invasive disease recurrence or death by 30% compared with standard endocrine monotherapy, with 3-year invasive disease-free survival rates of 92.4% and 89.6%, respectively [21]. Alongside these emerging endocrine strategies, CHT, alone or combined with ET, could provide clinical benefit in selected patients with higher baseline risk; however, the magnitude of such benefit varies according to individual factors and tumour biology [22].

In routine practice, the decision to use CHT is further shaped by age, comorbidities, frailty, patient preferences, and local care pathways. In addition, gene expression profiling is increasingly driving CHT decisions in hormone receptor-positive BC [23] Even when CHT is indicated, real-world use may differ from trial settings due to dose reductions, delays, early discontinuations, and complications requiring additional healthcare services [24]. These factors are particularly relevant in populations commonly underrepresented in clinical trials (e.g., older patients and those with multiple comorbidities) [25], where toxicity risk and supportive care needs may be substantial [26]. Therefore, describing high-risk ER+/HER2– eBC management in real clinical practice is important to understand treatment intensity and downstream healthcare burden. In this context, clinicopathological characteristics and patient-related factors may influence treatment selection and patterns of care, which in turn contribute to healthcare resource utilisation (HCRU) and associated direct costs [27].

From a health systems perspective, eBC generates a substantial long-lasting burden. Costs accrue from surgery, radiotherapy (RT), systemic therapies, surveillance, adverse event management, and recurrences, which can introduce further treatment needs and ongoing care [28]. Broad economic analyses have consistently reported that cancer management represents a major component of healthcare expenditures, with BC contributing significantly to overall costs and resource use in many countries [29,30]. While these estimates are informative, cost structures and treatment pathways differ between healthcare systems, making country-specific real-world evaluations essential, particularly in settings such as Italy, where the Italian National Health Service (INHS), a publicly funded system, is organised at regional level and Local Health Units (LHUs) are responsible for the provision and reimbursement of healthcare services, including hospitalisations, outpatient care, and pharmaceuticals. This organisational structure can shape treatment pathways, access to care, and cost distribution across regions.

Robust real-world evidence (RWE) can complement clinical trial findings by describing how treatments are used in clinical practice and what downstream resources and economic consequences are observed. In oncology, administrative healthcare databases are frequently employed to capture dispensed medications, hospitalisations, outpatient services, and reimbursed diagnostic procedures over time and across large populations, enabling assessments of HCRU and direct costs [27,31]. When linked to clinical sources such as pathology registries, these data can support more accurate identification of tumour subtype and high-risk features compared to claims data alone, which may lack granular clinical parameters [32]. This approach is particularly valuable in eBC, where laboratory and clinicopathologic information (e.g., ER/HER2 status, grade, nodal involvement details) substantially affects treatment selection and outcomes. Despite the importance of these matters, Italy-specific RWE remains limited, particularly regarding CHT utilisation patterns with the associated HCRU and direct economic burden in patients with high-risk disease. In this specific setting, indeed, treatment decisions are especially nuanced, and the downstream consequences of CHT-related toxicity, follow-up intensity, and recurrence risk may substantially affect the overall burden of care. Additionally, many published Italian RWE studies focus on metastatic disease [27], limiting current understanding of management pathways for high-risk ER+/HER2– eBC.

To address this gap, this real-world observational analysis was undertaken using linked administrative and pathological anatomy databases from the Italian healthcare system to generate evidence in patients with ER+/HER2– eBC, with a specific focus on the high-risk subgroup. A better understanding of treatment patterns and associated healthcare costs in this high-risk population is particularly relevant for healthcare planning and resource allocation within the Italian National Health Service, where evidence from routine clinical practice remains limited.

This analysis primarily aimed to describe the demographic and clinical characteristics of patients diagnosed with ER+/HER2– eBC and of those at high risk of recurrence, to evaluate treatment patterns in both populations, and to estimate the economic burden sustained by the INHS. Clinical outcomes, including recurrence rate, real-world event-free survival (rwEFS), and real-world overall survival (rwOS), were also assessed as exploratory objectives. The study was designed to provide descriptive estimates for the overall ER+/HER2– eBC population, with a specific focus on the high-risk subgroup.

## 2. Methods

### 2.1. Study Design and Data Sources

A retrospective, non-interventional cohort study was conducted using linked administrative databases and pathological anatomy databases of Italian LHUs, covering about 6 million health-assisted citizens. These LHUs, geographically distributed across the national territory, had data available from January 2010 to June 2024. Administrative databases capture information on healthcare services and resources reimbursed by the INHS, a system established in 1978 that ensures universal coverage for all residents. For this analysis, the following databases were used: a beneficiary database for demographic information (sex, age, and date of death, if applicable); a pharmaceutical claims database, reporting INHS-reimbursed medications, with their Anatomical Therapeutic Chemical (ATC) code, dispensing date, number of packages, units per package, and unit cost per package; a hospitalisation database containing inpatient admissions, namely primary/secondary discharge diagnoses coded through the International Classification of Diseases, Ninth Revision, Clinical Modification (ICD-9-CM, namely the official coding system used for Italian hospital discharge records throughout the study period), the Diagnosis-Related Group (DRG), and the corresponding DRG-based reimbursement paid by the INHS; an outpatient specialist service database, including reimbursed visits and diagnostic procedures (date, type of service, description, and tariff/charge for laboratory tests or specialist consultations); and an exemption database recording active exemption codes that waive patient co-payments for services and treatments related to specific diagnosed conditions.

The pathological anatomy database provided histopathological examination information to support clinical characterisation, including biomarker status and staging elements.

The dataset used consists solely of anonymised data, as defined in accordance with applicable regulations. All the results of the analyses were produced and presented as aggregated summaries. This analysis was conducted in accordance with the principles of the Declaration of Helsinki. Approval has been obtained from the ethics committees of the participating healthcare entities.

The analysis results are reported exclusively in an aggregate format; consequently, they cannot be attributed, either directly or indirectly, to any individual patient.

In line with the risk-based criteria set out in Opinion 05/2014 on Anonymisation Techniques of the Article 29 Data Protection Working Party, and as a precautionary disclosure-control measure, analyses involving fewer than or equal to three patients were not reported. Accordingly, analysis results referring directly or indirectly to fewer than or equal to three patients were flagged as NI (not issuable).

### 2.2. Patient Population and Timeline

Considering the overall period of data availability (January 2010–June 2024), the inclusion period was defined as the entire timeframe covered by the pathological anatomy databases, which in most participating LHUs largely overlapped with the availability of administrative healthcare data. The index-date was defined as the first recorded date of BC-related surgery within the inclusion period. Patients were characterised during a baseline period corresponding to all available information prior to the index-date, with a minimum look-back of 12 months, to assess prior treatments, comorbidities, hospitalisations, diagnostic procedures, and outpatient specialist services. Follow-up extended from the index-date to the end of data availability, with at least 12 months required for recurrence analyses. Of note, as patients entered the cohort at different times during the study period, the duration of potential follow-up varied according to the index year. Time-to-event analyses accounted for differences in observation time through censoring at the end of data availability.

Patients with BC were initially screened from LHUs’ databases using the following criteria: adult women (≥18 years) with evidence of BC in hospital discharge records, defined as at least one discharge diagnosis coded as ICD-9-CM 174, and with a record of surgical treatment for BC, defined as at least one procedure coded as ICD-9-CM 85.2–85.4. Patients were excluded if they were younger than 18 years, male, had received ET or CDK4/6i in the neoadjuvant setting, had a recent history of another primary malignancy within the prior two years, or had no record of BC surgery.

### 2.3. Identification of ER+/HER2– Status

ER+/HER2– status was ascertained using both administrative and pathological anatomy sources. Within administrative databases, HER2 status was defined according to the presence (HER2+) or absence (HER2–) of exposure to HER2-targeted agents (ATC codes L01XC03, L01FD01, L01XY02, L01XC13, L01FD02, L01FD03, L01FD05, L01XE07, L01EH01, L01XE45, L01EH02, L01EH03) over the entire available study period. ER positivity was identified based on evidence of ET, defined as prescriptions for tamoxifen (ATC code L02BA01), or aromatase inhibitors, including anastrozole (L02BG03), letrozole (L02BG04), and exemestane (L02BG06). ER positivity was also identified by the presence of an ICD-9-CM code indicating ER-positive status (V86.0), assessed across the full observation window. Within the pathological anatomy database, HER2 negativity was identified through text-based searches capturing the presence of the strings “HER–2” or “HER2” in combination with negativity indicators (e.g., “NEG*” or “–”); ER positivity was identified through the presence of key substrings such as “ER” or OESTROGEN* and positivity indicators (e.g., “ER” and “+”).

### 2.4. Identification of eBC Status and the High-Risk ER+/HER2– eBC Subgroup

Likewise, eBC status and the high-risk ER+/HER2– eBC subgroup were defined using a composite approach combining administrative and pathological information. First, eBC (non-metastatic) status was operationally identified based on the absence of documented metastatic disease, assessed in the pathological anatomy database by the absence of the substring “M1” and supported in administrative data by the absence of hospital discharge diagnoses for metastases (ICD-9-CM 196–198) and/or the absence of prescriptions consistent with management of bone metastases (ATC codes M05BA08, 041300, M05BA03) from 2017 onwards. This operational definition did not necessarily imply pathologically confirmed M0 status in all women. Patients with evidence of CDK4/6i use were excluded, based on prescriptions for palbociclib (ATC L01EF01), ribociclib (L01EF02), or abemaciclib (L01EF03). From June 2023 onwards, abemaciclib prescriptions were not considered an exclusion criterion when consistent with adjuvant use, following its approval in the adjuvant setting by the Italian Medicines Agency (AIFA) [33].

Then, high-risk ER+/HER2– eBC was identified among patients with Grade 3 tumours, based on clinical TNM stage patterns consistent with higher-risk presentations, namely clinical stage T1c–T2 with N1–cN2, or T3–T4 with N0–N2; when TNM components were unavailable, stage group II or III was used, where reported, to classify patients as high-risk, consistent with KEYNOTE-756 [34]. Similar clinicopathological criteria have also been adopted in a US real-world study of patients with high-risk ER+/HER2– eBC, supporting the clinical relevance of this approach for identifying a selected high-risk population [21]. However, the use of stage II–III disease when individual TNM components were unavailable represented a pragmatic adaptation to the information available in routine clinical records, rather than an exact replication of KEYNOTE-756 eligibility criteria [34].

### 2.5. Baseline Demographic and Clinical Characteristics

Baseline demographic and clinical features were assessed during the characterisation period preceding the index-date, using at least 12 months of available data. The evaluation included age at surgery and the general comorbidity burden, which was measured through a modified version of the Charlson Comorbidity Index (CCI) that excluded the weighting of cancer [35]. In addition, the presence of specific conditions such as cardiovascular disease, diabetes, hyperlipidaemia, neuropathy, and liver disorders was investigated during the entirety of the available pre-index characterisation period. Comorbidities were identified through diagnosis codes and exemption records, while medication use was detected using the ATC codes.

### 2.6. Treatments and Procedures

Surgical procedures were captured through hospitalisation records and classified as either breast-conserving surgery or mastectomy. Neoadjuvant therapy was evaluated during the six months preceding surgery. Exposure to CHT was determined from pharmacy and hospitalisation data, while neoadjuvant RT was identified through outpatient procedure claims. Patients who received ET and CDK4/6i before surgery were excluded from this study.

The adjuvant period was assessed considering the time from surgery until metastatic diagnosis or to the end of follow-up. Systemic treatments, including CHT, ET, CDK4/6i (abemaciclib only, after its approval) and combined CHT plus ET regimens, were identified through pharmacy dispensations and hospital or outpatient service data. RT administered after surgery was also recorded. Patients who received CHT both before and after surgery were identified separately to describe more intensive treatment patterns.

Lymph node biopsy and lymph node dissection were evaluated during the six months before and after surgery using procedure codes.

### 2.7. Healthcare Resource Utilisation and Direct Costs

HCRU and direct healthcare costs were calculated during the neoadjuvant phase (6 months before surgery), during the whole available follow-up (annualised) and in the five years after surgery on a yearly basis. Post-surgery assessments included patients while they remained alive and recurrence-free, with follow-up censored at recurrence, death, or the end of data availability. Accordingly, these analyses were designed to estimate the economic burden of eBC management during the recurrence-free period, rather than costs attributable to recurrent/metastatic disease or end-of-life care.

HCRU was assessed through administrative claims for hospital admissions, outpatient procedures, and drug dispensations. Cost analyses were conducted from the perspective of the INHS and presented in euros (€). Costs were inflation-adjusted to 2024 € based on the healthcare Consumer Price Index (CPI). Hospitalisation costs were estimated using DRG reimbursements, outpatient services were calculated according to regional tariffs, and medication costs were calculated by multiplying each dispensed unit by its corresponding reimbursement value. Extreme cost outliers, namely patients with costs exceeding the mean by more than three standard deviations (SD), were excluded from cost analyses. This conventional statistical threshold was adopted to identify observations markedly distant from the mean and limit their disproportionate influence on descriptive average cost estimates.

### 2.8. Outcome Definitions

For the purposes of this analysis, recurrence was operationally defined, per clinical input, as the earliest occurrence of metastatic disease based on discharge hospitalisation diagnoses for metastases (ICD-9-CM codes: 196.1, 196.2, 196.3, 196.4, 196.5, 196.6, 196.9, 197.3, 197.4, 197.5, 197.6, 197.7, 198.0, 198.1, 198.3, 198.4, 198.5, 198.6, 198.7, 198.2, 198.81, 198.82, 198.89, 199.0), initiation of medications used in the management of bone metastases, or initiation of CDK4/6i before June 2023, the time of abemaciclib approval in the adjuvant setting. This proxy-based definition primarily captured recurrence events associated with metastatic disease or changes in systemic treatment, whereas local recurrences managed exclusively with surgery or radiotherapy could not be reliably identified through the available administrative data. Accordingly, this outcome should be interpreted primarily as a proxy for metastatic/distant recurrence rather than as a comprehensive measure of all BC recurrences. Only patients with at least 12 months of follow-up after surgery were included in recurrence analyses. Time to recurrence was calculated through Kaplan–Meier survival analysis for both the ER+/HER2– eBC cohort and the high-risk subgroup. Recurrence rates were expressed as the number of events per 1000 person-years. Cumulative rates were calculated using the total number of events and person-time accrued from the index-date up to each follow-up time point, whereas annual rates referred to individual yearly intervals. These measures were distinguished from Kaplan–Meier estimates of recurrence-free probability.

rwEFS was defined as the time from the index-date to the first occurrence of recurrence, disease progression requiring systemic treatment, or death. Patients without an event were censored at the end of follow-up. Estimates of one- to four-year survival were generated with the Kaplan–Meier method and evaluated separately for the overall and high-risk cohorts.

Lastly, rwOS was defined as the time from the index-date to death from any cause, based on information from the beneficiary registry. Patients without a death record were censored at the last date of data availability. Kaplan–Meier curves were used to estimate survival at one, two, three, and four years post-surgery for both cohorts.

### 2.9. Statistical Analysis

Demographic, clinical, and economic data were analysed descriptively. Categorical variables were reported as frequencies and percentages, and continuous variables were summarised as mean ± SD, median, interquartile range (IQR; Q1–Q3), and range (minimum–maximum), as appropriate. The study was designed as a descriptive analysis of the overall ER+/HER2– eBC population (Cohort #1) and the high-risk subgroup identified within this population (Cohort #2). As Cohort #2 was a subset of Cohort #1, the two populations were not mutually exclusive. Estimates were reported separately to characterise the high-risk subgroup within the broader study population, without formal between-group comparisons or adjustment for potential confounders. Accordingly, differences in descriptive estimates should not be interpreted as independent associations with high-risk status.

Time-to-event clinical outcomes (time to recurrence, rwEFS, and rwOS) were analysed using the Kaplan–Meier method, with censoring at the last available follow-up for patients without an event. Kaplan–Meier estimates were generated separately for the overall population and the high-risk subgroup to describe their respective event and survival probabilities over time, without adjustment for baseline demographic or clinical characteristics.

All the analyses were performed using Stata SE version 17.0 (StataCorp, College Station, TX, USA).

## 3. Results

### 3.1. Study Population and Cohort Selection (January 2010–June 2024)

**Figure 1:**
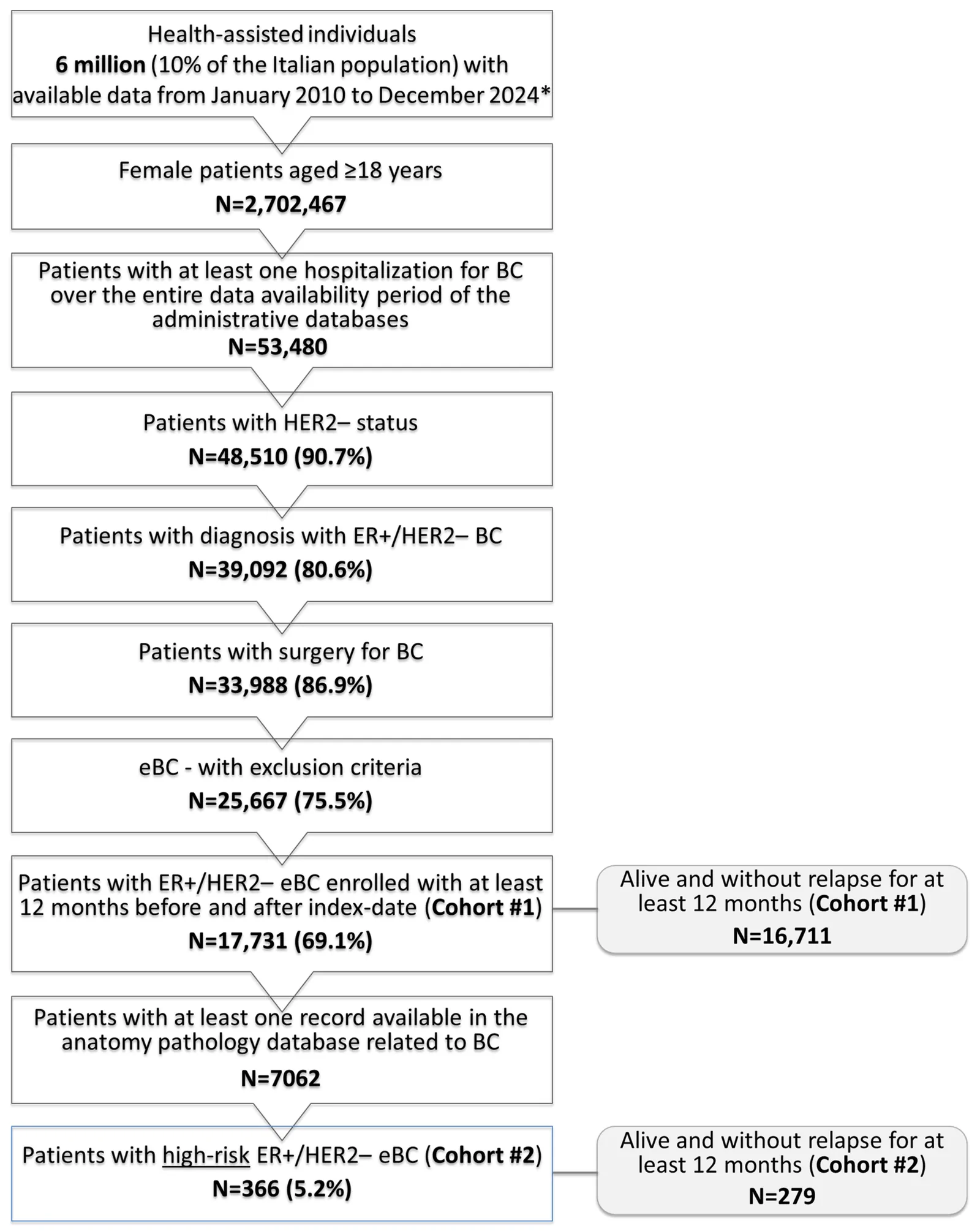
Flowchart of patient selection. * Depending on data availability for each LHU involved (for some LHUs the period of data availability started later than January 2010 and/or ended before June 2024). No history of another primary cancer during the 24 months before the index-date and no use of ET before surgery. *Abbreviations*: BC, breast cancer; eBC, early breast cancer; ER+, oestrogen receptor-positive; HER2–, human epidermal growth factor receptor 2-negative.

Among women identified with BC in the source population during the inclusion period, 17,731 constituted Cohort #1 after applying the inclusion/exclusion criteria and requiring at least 12 months of continuous data before and after the index-date. Within Cohort #1, 366 patients were classified as high-risk ER+/HER2– eBC, constituting Cohort #2. For economic and clinical outcome analyses, the corresponding recurrence-free populations included 16,711 patients in Cohort #1 and 279 patients in Cohort #2. The sequential steps of cohort selection are detailed in Figure 1.

The length of the characterisation period was 5.3 ± 2.8 (median 5.0; IQR: 3.9) years in Cohort #1 and 4.9 ± 2.3 (median 4.9; IQR 3.9) years in Cohort #2. The length of the follow-up was 5.1 ± 2.8 (median 4.8; IQR: 4.1) years in Cohort #1 and 4.0 ± 2.1 (median 3.7; IQR 3.3) years in Cohort #2.

### 3.2. Baseline Demographic and Clinical Profile of the Study Population

Baseline demographic and clinical profiles of women in the two cohorts are detailed in Table 1. Mean age was 62.7 years in the overall ER+/HER2– eBC population and 66.9 years in the high-risk subgroup: 46.2% of the overall patients were aged ≥65 years; in the high-risk subset, this proportion was 58.2%. Mean CCI values were 0.3 in Cohort #1 and 0.4 in Cohort #2. In the overall population, cardiovascular comorbidities, diabetes, and hyperlipidaemia were recorded in 44.7%, 9.8%, and 18.2% of the patients; in the high-risk subgroup the corresponding proportions were 53.3%, 14.2%, and 23.0%, respectively. In the 12 months preceding surgery, the mean number of total filled prescriptions (both oncological and non-oncological) was 5.5 in the overall population and 6.2 per patient in the high-risk subgroup.

### 3.3. Treatment Patterns

Table 2 summarises the surgical procedures and systemic/radiation treatments received before and after surgery by patients in Cohort #1 (ER+/HER2– eBC, N = 17,731) and Cohort #2 (high-risk ER+/HER2– eBC, N = 366). All the patients underwent surgery in both cohorts, by design. Breast-conserving surgery was the most common procedure overall, accounting for 80.6% of procedures in Cohort #1 and 53.6% in Cohort #2, while mastectomy accounted for 19.4% and 46.4%, respectively.

In the neoadjuvant setting, defined here as treatment administered before surgery, therapy was recorded in 709/17,731 patients (4.0%) in Cohort #1 and 40/366 patients (10.9%) in Cohort #2. Neoadjuvant CHT accounted for the largest share of neoadjuvant treatment in Cohort #1 (699/709, 98.6%) and total neoadjuvant treatment in Cohort #2 (40/40, 100%). Neoadjuvant RT was rare and observed only in Cohort #1 (12/709, 1.7%).

In the adjuvant setting, defined here as treatment administered after surgery before metastasis diagnosis, 16,827 patients (94.9%) in Cohort #1 and 306 patients (83.6%) in Cohort #2 received at least one adjuvant treatment. Adjuvant CHT was recorded in 356/16,827 patients (2.1%) of Cohort #1 and 61/306 patients (19.9%) of Cohort #2. Combined adjuvant CHT plus ET was detected in 3045/16,827 patients (18.1%) and 106/306 patients (34.6%). Overall, total documented ET use, including ET alone and combined CHT plus ET, was 96.5% in Cohort #1 and 77.4% in Cohort #2. The lower-than-expected ET rate observed in Cohort #2 should be interpreted with caution, as ET use may be under-ascertained in administrative databases, particularly for outpatient and long-term treatments. CHT in both the neoadjuvant and adjuvant settings was found in 183 of the total 17,731 patients (1.0%) in Cohort #1 and 14 of 366 patients (3.8%) in Cohort #2.

Overall, descriptive findings characterise the surgical and systemic treatment patterns observed in the high-risk subgroup within the broader ER+/HER2– eBC population. In Cohort #2, lymph node removal within six months after surgery was recorded in 36.3% of patients, and lymph node biopsy in 7.9%, while the corresponding proportions in Cohort #1 were 12.6% and 2.2%, respectively.

### 3.4. Healthcare Resource Consumption and Direct Costs During the Neoadjuvant Phase

During the pre-index neoadjuvant phase, HCRU was described separately for the overall population and the high-risk subgroup. In both cohorts, resource consumption was mainly driven by pharmacy claims and outpatient services rather than direct CHT- or RT-related care. Hospitalisations were infrequent, while CHT- and RT-related claims accounted for only a small share of overall resource use (Table 3).

As detailed in Table 4, during the neoadjuvant period (6 months before surgery), the mean cost per patient in the overall cohort (N = 17,399) was €906.1, with outpatient services representing the largest share (€482.0), followed by medications and hospitalisation (respectively €254.9 and €169.2). In the high-risk cohort (N = 355), the mean cost during the neoadjuvant phase was €1377.2, primarily due to outpatient services (€729.0), followed by drug (€379.9) and inpatient expenses (€268.2). Overall, costs were mainly driven by outpatient services in both cohorts. In the high-risk cohort, outpatient services remained the largest cost component, but hospitalisation and medication costs also contributed substantially to the overall economic burden. In both cohorts, expenditures were largely attributable to services and treatments not directly related to CHT or RT.

### 3.5. Annual Healthcare Resource Consumption and Direct Costs During Follow-Up (Adjuvant Therapy)

Table 5 presents annualised HCRU estimates for the overall ER+/HER2– eBC population and the high-risk subgroup. In Cohort #2, the mean (± SD) numbers of total claims and pharmacy claims per patient were 30.3 ± 14.2 and 17.3 ± 10.0, respectively; the corresponding estimates in Cohort #1 were 26.3 ± 12.5 and 15.4 ± 8.8. The mean number of drugs used in the adjuvant regimen was 4.9 ± 3.3 in Cohort #2 and 5.6 ± 2.8 in Cohort #1, while other pharmacy claims averaged 12.5 ± 9.8 and 9.8 ± 8.2 per patient, respectively.

In Cohort #2, the mean number of hospitalisation claims was 0.9 ± 0.9 per patient, including surgery-related admissions (0.5 ± 0.4) and other hospitalisations (0.5 ± 0.6). The corresponding estimates in Cohort #1 were 0.6 ± 0.7, 0.3 ± 0.3, and 0.3 ± 0.6, respectively. Outpatient service claims averaged 12.0 ± 7.5 in Cohort #2 and 10.3 ± 6.9 in Cohort #1, including CHT-related claims (0.5 ± 1.5 and 0.2 ± 1.3) and RT-related claims (0.7 ± 1.6 and 0.7 ± 1.8), respectively.

Table 6 presents the annual direct healthcare costs per patient during follow-up for the overall population with ER+/HER2– eBC (Cohort #1) and for the high-risk subgroup (Cohort #2), excluding deaths and cost outliers.

Overall, the total annual mean cost per patient was €4671.4 in Cohort #1 (N = 16,696), whereas the corresponding estimate was €6934.0 in Cohort #2 (N = 290). Pharmacy costs averaged €1374.2 in the overall cohort and €1512.7 in the high-risk subgroup. Within this category, drugs used in the adjuvant regimen accounted for €509.2 in Cohort #1 and €589.7 in Cohort #2, while other drug-related costs were €865.0 and €923.0, respectively.

Hospitalisation costs represented a major component of annual direct costs, particularly in the high-risk subgroup. Mean annual hospitalisation costs were €1838.4 in Cohort #1 and €3658.1 in Cohort #2. Surgery-related costs amounted to €987.5 in the overall population and €1547.7 in high-risk patients, while other (non-oncological) hospitalisation costs were €850.9 and €2110.4, respectively.

Outpatient service costs were also reported separately for the two populations, with mean values of €1458.8 in Cohort #1 and €1763.2 in Cohort #2. CHT-related outpatient costs were €27.5 in Cohort #1 and €147.6 in Cohort #2, while the corresponding RT-related costs were €692.9 and €614.5, respectively. In summary, the descriptive analysis documented a substantial annual direct healthcare burden in the high-risk ER+/HER2– eBC subgroup, with hospitalisations representing the largest cost component, followed by outpatient services and pharmaceuticals.

### 3.6. Clinical Outcomes

**Figure 2:**
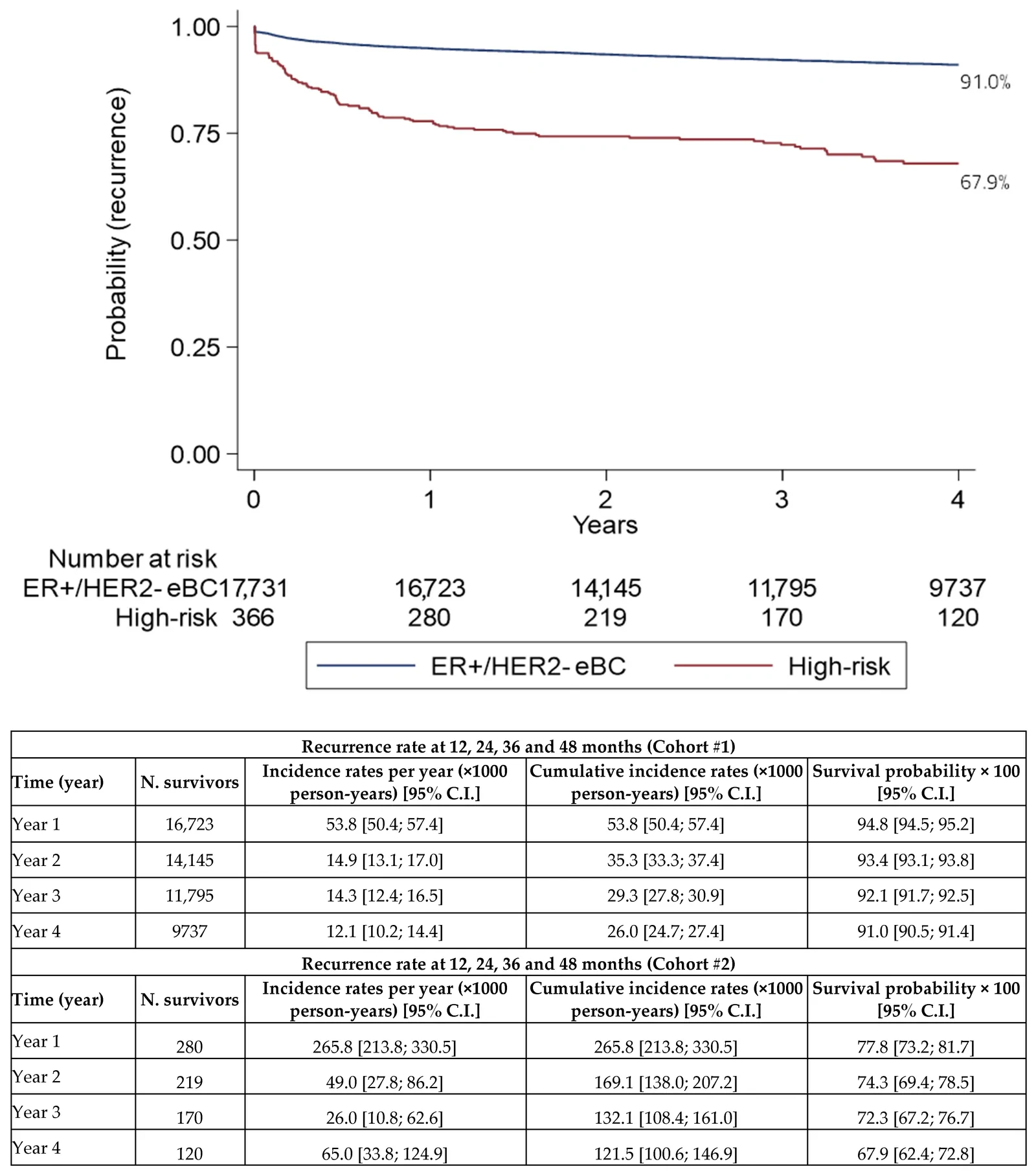
Recurrence rate at 1, 2, 3, and 4 years among patients with ER+/HER2– eBC (Cohort #1) and patients with high-risk ER+/HER2– eBC (Cohort #2). *Abbreviations*: CI, confidence interval; eBC, early breast cancer. Note: Cumulative follow-up incidence rates were calculated per 1000 person-years and may decrease over time, reflecting fewer events during later follow-up.

Clinical outcomes were described for the overall ER+/HER2– eBC population and separately for the high-risk subgroup. Regarding recurrence rate, in the overall cohort (Cohort #1), cumulative event rates (per 1000 person-years) were 53.8 at 1 year, 35.3 at 2 years, 29.3 at 3 years, and 26.0 at 4 years after surgery. In the high-risk subgroup (Cohort #2), the corresponding estimates were 265.8 (per 1000 person-years) at 1 year, 169.1 at 2 years, 132.1 at 3 years, and 121.5 at 4 years (Figure 2). These estimates represent events per unit of accumulated person-time rather than cumulative probabilities of recurrence.

**Figure 3:**
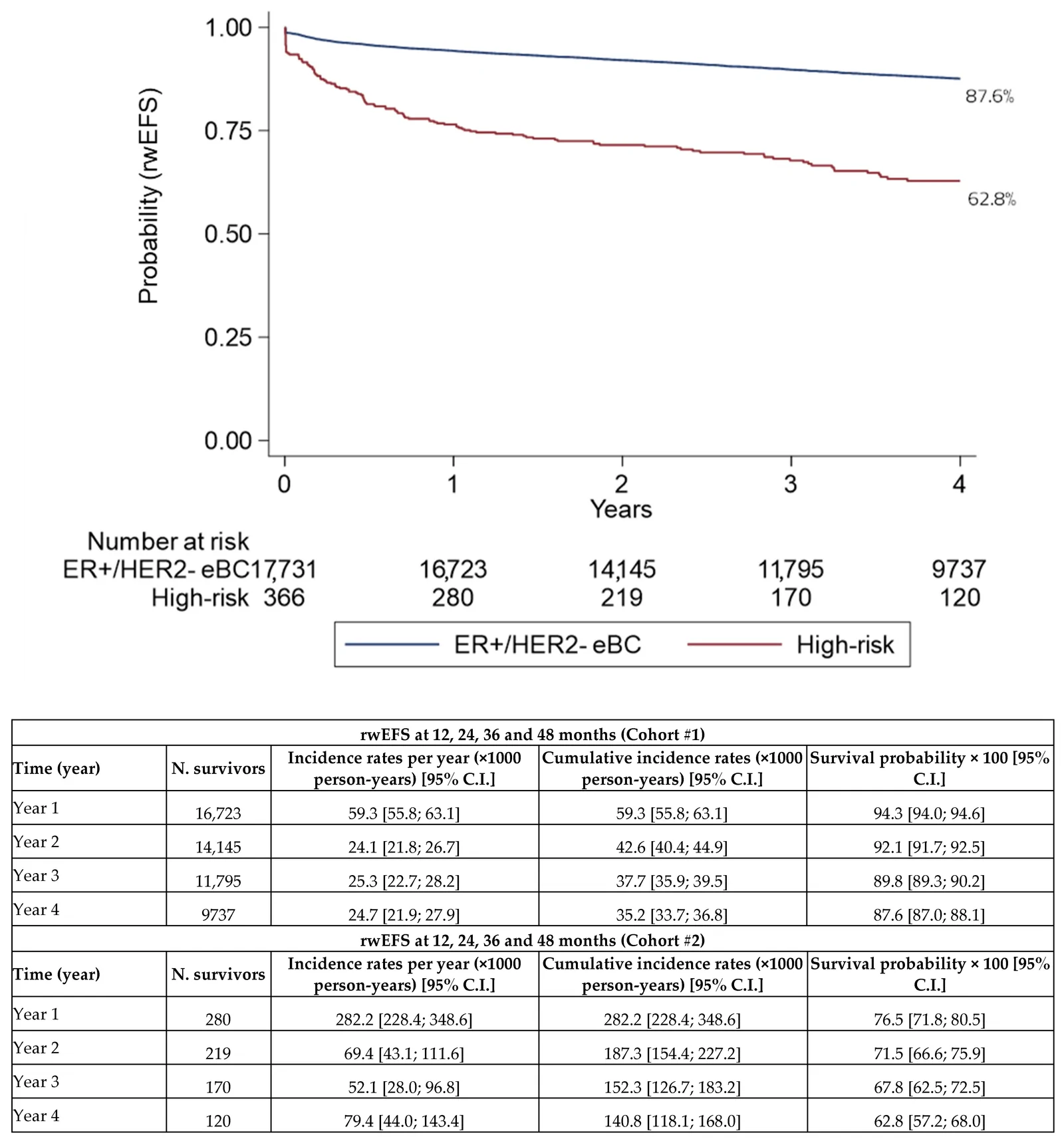
rwEFS at 1, 2, 3, and 4 years among patients with ER+/HER2– eBC (Cohort #1) and patients with high-risk ER+/HER2– eBC (Cohort #2). *Abbreviations*: CI, confidence interval; eBC, early breast cancer; rwEFS, real-world event-free survival.

The rwEFS analysis provided additional descriptive information on clinical outcomes. In Cohort #1, rwEFS remained high, ranging from 94.3% at year 1 to 87.6% at year 4. Among patients in Cohort #2, rwEFS probability was 76.5% at 1 year, 71.5% at 2 years, 67.8% at 3 years, and 62.8% at 4 years (Figure 3).

**Figure 4:**
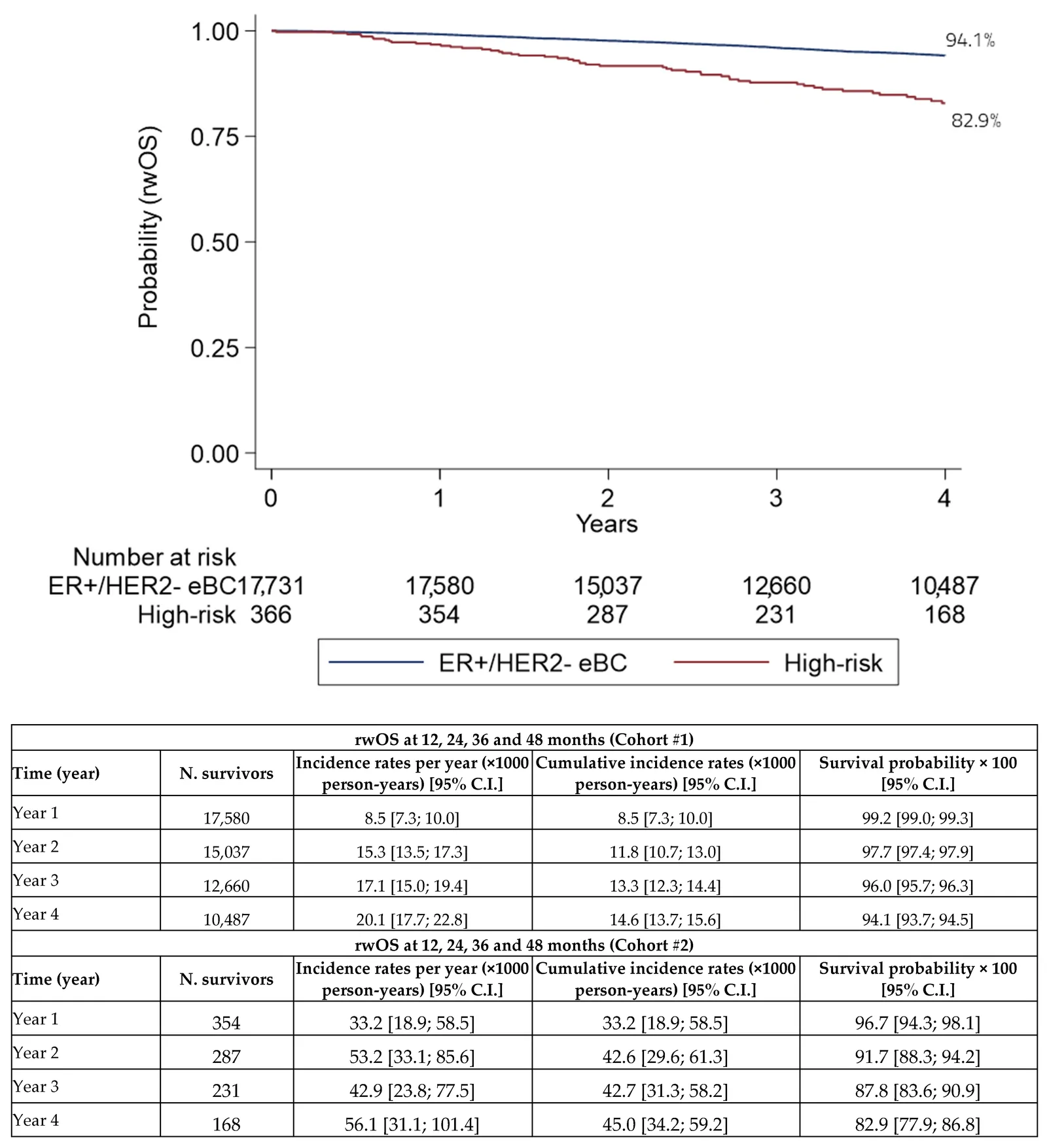
rwOS at 1, 2, 3, and 4 years among patients with ER+/HER2– eBC (Cohort #1) and patients with high-risk ER+/HER2– eBC (Cohort #2). *Abbreviations*: CI, confidence interval; eBC, early breast cancer; rwOS, real-world overall survival.

Survival outcomes over the first four years after surgery were favourable in the overall ER+/HER2– eBC cohort, in line with expectations for early-stage disease managed with curative intent. In Cohort #1, rwOS remained above 99% at the first year and exceeded 94% at the fourth year. In the high-risk subgroup, estimated rwOS was 96.7% at 1 year, 91.7% at 2 years, 87.8% at 3 years, and 82.9% at 4 years (Figure 4).

## 4. Discussion

This retrospective RWE analysis linked administrative claims with pathology databases from Italian LHUs to characterise treatment patterns, HCRU, costs, and outcomes in women with ER+/HER2– eBC, with a specific focus on the high-risk subgroup. The descriptive analysis characterised the demographic and clinical profile of these patients, together with patterns of locoregional and systemic management, including mastectomy, axillary procedures, and CHT. The findings also documented substantial healthcare resource consumption and direct costs, along with considerable recurrence risk and reduced rwEFS and rwOS estimates within the high-risk subgroup. The observations highlight the clinical and economic burden of high-risk ER+/HER2– eBC in routine practice. Surgical locoregional treatment patterns were notably more extensive in the high-risk cohort, with mastectomy performed in more than twice as many patients compared with the overall cohort, and post-surgical lymph node removal occurring at nearly three times the rate observed in the broader population. In the high-risk subgroup, surgical management included mastectomy in 46.4% of patients and post-surgical lymph node removal in 36.3%. These patterns may reflect the adverse clinicopathological characteristics included in the high-risk definition, such as Grade 3 disease, larger tumour size, and nodal involvement, which are relevant factors in surgical decision-making. Importantly, contemporary practice has moved toward de-escalation of axillary surgery in selected patients to reduce morbidity (e.g., lymphedema) while preserving outcomes. However, de-escalation is less applicable when nodal burden is high or when staging information is incomplete, a situation that can occur in routine care [36]. In this context, the observed use of axillary procedures in the high-risk subgroup may reflect surgical decision-making in patients with adverse clinicopathological characteristics or incomplete staging information.

Regarding systemic therapy, CHT-based strategies were documented in both the neoadjuvant and adjuvant settings among patients meeting the predefined high-risk criteria, providing insight into treatment patterns in this clinically selected subgroup. RT use should be interpreted in the context of surgical management: breast-conserving surgery is generally followed by adjuvant RT, whereas RT after mastectomy is reserved for selected patients with higher-risk features, such as nodal involvement or larger tumour burden, to optimise locoregional control [37]. Therefore, the observed RT patterns should be interpreted in the context of the surgical procedures documented in each population, particularly the use of breast-conserving surgery and mastectomy. This distribution aligns with international consensus and guideline frameworks in which higher clinical risk and aggressive pathology increase the likelihood of recommending CHT in addition to ET, and support tailored use of RT according to surgery type and locoregional recurrence risk [38].

Although only some of the key clinicopathologic features could be captured through linkage between administrative claims and pathology databases, the observed pattern is consistent with St. Gallen consensus principles emphasising escalation for patients with adverse clinicopathologic features, while tailoring treatment intensity using biomarkers and multigene assays where appropriate [39]. At the same time, the relatively low ET-alone proportion in the high-risk subgroup could be due to incomplete ET capture in linked datasets when prescriptions occur outside reimbursed pathways or when follow-up is truncated; nevertheless, this analysis required continuity of data, likely mitigating (though not completely eliminating) this issue. This potential under-ascertainment of ET may also have led to an underestimation of drug-related costs in Cohort #2. More specifically, the lower-than-expected ET use may reflect incomplete capture of long-term oral treatments rather than actual undertreatment or poor adherence. Therefore, the observed ET rates should not be interpreted as direct indicators of treatment adherence. In addition, incomplete ET ascertainment may lead to an underestimation of ET-related pharmaceutical expenditures and, consequently, of overall adjuvant treatment costs. Nevertheless, since hospitalisations represented the main cost driver, this limitation is unlikely to fully explain the elevated overall healthcare costs observed in the high-risk cohort.

Regarding CHT, our findings provide descriptive information on the use of CHT-based strategies in the high-risk subgroup. These treatment patterns may be consistent with risk-adapted clinical practice, although concordance with treatment guidelines cannot be definitively assessed without multigene assay results and detailed staging information for all patients. In the broader literature, multigene assays have reduced CHT use in clinically intermediate-risk populations by identifying patients with limited incremental benefit from CHT, as shown in pivotal trials, especially in node-negative disease [23]. Conversely, in node-positive postmenopausal patients, RxPONDER supports sparing CHT in selected patients while emphasising benefit in premenopausal patients, underscoring how age/menopausal status and biology interact with clinical risk [40].

The economic analysis is consistent with a previous Italian report describing the characteristics, management, and direct healthcare costs of patients with HR+/HER2− eBC using a similar real-world approach based on linked administrative and pathology databases [27]. The study confirmed that HR+/HER2− patients represent the majority of eBC cases and are associated with substantial HCRU and costs, particularly for hospitalisations and systemic treatments. Furthermore, the authors highlighted variability in treatment patterns and resource consumption across patient subgroups, suggesting that clinical risk stratification drives both management decisions and costs [27]. These treatment patterns may be consistent to risk-adapted clinical practice, although concordance with treatment guidelines cannot be definitively assessed without multigene assay results and detailed staging information for all patients. The annualised follow-up data documented substantial HCRU and direct costs among high-risk patients, reflecting the burden of locoregional management, systemic treatment, and ongoing healthcare needs in this specific population. Importantly, while hospitalisations were less frequent than outpatient services, they remain a key cost driver. This aligns with data from a multinational study in the European Union indicating that inpatient episodes, particularly those related to surgery and complications, typically represent major components of healthcare expenditures attributable to cancer [29]. Other European real-world studies have similarly shown that HR+/HER2− disease, despite generally more favourable prognosis, still accounts for a large share of total BC-related costs due to its high prevalence and long treatment duration, reinforcing its relevance from the healthcare system perspective [41,42,43]. In our analysis, the high-risk subgroup showed a substantial healthcare burden, with hospitalisations representing a major component of direct costs. These findings are also relevant from an outcome perspective, as recurrence and progression are known to increase downstream costs through more intensive systemic therapy, outpatient care, and hospital use [44]. Consistently, a recent US real-world study specifically focusing on high-risk ER+/HER2– eBC documented substantially greater cost burden among patients experiencing recurrence than among those without recurrence [45]. In Italy, administrative data similarly identified hospital care, outpatient services, and drug prescriptions are key drivers of BC expenditures [46].

Over the 4-year follow-up, rwOS exceeded 94% in the overall ER+/HER2– eBC population. Within the high-risk subgroup, recurrence and rwEFS events were observed throughout follow-up, and rwOS was estimated at 82.9% at 4 years. These descriptive findings characterise the clinical burden among patients meeting the predefined high-risk criteria, although the contribution of individual clinicopathological factors could not be independently assessed. These findings are coherent with the natural history of ER-positive disease, which is characterised by risk of recurrence that may persist for more than 10 years after diagnosis [10]. Since the present follow-up captures medium-term outcomes, longer observation would be required to fully quantify the late recurrence rates, particularly in the overall cohort where events may accumulate more gradually over time [34]. The evolving therapeutic landscape may also influence future HCRU and direct healthcare costs in Italy. The introduction of adjuvant CDK4/6i, including abemaciclib and ribociclib, may increase pharmaceutical expenditures and monitoring requirements. Similarly, the potential incorporation of immunotherapy-based strategies into routine clinical practice could further increase treatment-related resource utilisation. However, improvements in clinical outcomes may help reduce recurrence-related healthcare costs over the longer term. The overall economic impact of these strategies will depend on treatment uptake, long-term effectiveness, and reimbursement policies within the INHS, highlighting the need for future real-world evaluations. The high-risk definition adopted here likely captures patients with both early and persistent recurrence risk. Moreover, recurrence was identified through administrative proxies; while suitable for large-scale RWE analyses, this approach may be affected by changes in care trajectories over longer follow-up.

Key strengths of this RWE analysis include the use of linked pathology and administrative claims data, which improved cohort definition and enabled comprehensive capture of treatments, HCRU, and reimbursed costs in routine practice. The inclusion of LHUs geographically distributed across the Italian territory, together with longitudinal follow-up, supports robust real-world estimates of clinical outcomes alongside healthcare burden. Building on these strengths, the present findings provide a foundation for future research aimed at further characterising the long-term clinical and economic burden of high-risk ER+/HER2– eBC in routine practice. Analyses with longer follow-up could provide additional insights into recurrence patterns, survival outcomes, and associated healthcare costs over time. Moreover, integrating administrative data with more detailed clinical and molecular information could improve risk stratification and support a more comprehensive assessment of treatment effectiveness. Future research should also evaluate the clinical and economic impact of emerging therapeutic strategies in this population.

On the other hand, some limitations should be considered when interpreting the results. The study was designed as a descriptive analysis, and the high-risk cohort was a subset of the overall ER+/HER2– eBC population rather than an independent comparison group. No formal between-group comparisons or adjusted analyses were performed; therefore, differences in demographic and clinical characteristics, treatment exposure, and calendar period may have influenced the findings, which should not be interpreted as independent associations with high-risk status. Future studies using mutually exclusive cohorts and adjusted analyses could address these limitations.

Another limitation concerns the identification of biomarkers and staging components through pathology text parsing and administrative treatment proxies, potentially introducing misclassification and influencing treatment pattern estimates. The absence of pathology-confirmed sensitivity analyses further restricts the assessment of these definitions. Similarly, the absence of documented metastatic disease did not necessarily confirm M0 status, while the use of stage II–III disease when complete TNM information was unavailable may have introduced further misclassification of high-risk status.

The limited size of the high-risk subgroup, consistent with previous real-world findings [21], precluded further stratification by tumour size, nodal status, and other prognostic factors. Larger cohorts and validated classification criteria could improve the robustness and granularity of future analyses.

Recurrence and rwEFS were based on administrative proxies (metastasis codes, bone-targeted drugs, and CDK4/6i timing assumptions), which may under- or overestimate clinical events. In particular, local recurrences managed exclusively with surgery or radiotherapy may have been missed, potentially leading to an overestimation of recurrence-free survival and rwEFS. Moreover, death was not accounted for as a competing event in recurrence analyses, and Kaplan–Meier estimates may consequently overestimate recurrence probability. The absence of formal algorithm validation, sensitivity analyses using alternative definitions, and competing-risk methods further limits the robustness of these estimates.

Regarding economic outcomes, post-surgery costs were assessed only during recurrence-free follow-up, excluding expenditures associated with recurrent/metastatic disease and end-of-life care. Consequently, they do not represent the total economic burden of high-risk ER+/HER2– eBC.

Furthermore, therapeutic regimen information and related costs, especially for CHT, were incompletely captured. Administrative databases may lack information on clinical intent, dosing, privately provided care, and specific CHT agents administered in outpatient settings. In addition, indirect and societal costs, including productivity losses, caregiver burden, transportation expenses, and out-of-pocket expenditures, were not captured, as the analysis was conducted from the INHS perspective. Lastly, the exclusion of cost outliers exceeding the mean by more than three SD may have led to an underestimation of healthcare expenditures, particularly among high-risk patients. The absence of sensitivity analyses retaining these observations limits the assessment of their impact on the reported cost estimates.

Similarly, incomplete ascertainment of long-term oral ET may have affected treatment and pharmaceutical cost estimates. As dispensing records do not confirm medication intake, observed ET rates should not be interpreted as measures of adherence. Integrating additional clinical and prescribing information could improve these estimates.

Another aspect to consider is the extended observation period (2010–2024), during which diagnostic approaches, treatment strategies, clinical guidelines, and reimbursement policies evolved considerably. Although differential follow-up was addressed through censoring, patients included in more recent years had shorter observation periods, while the absence of stratification or adjustment by calendar period limits the assessment of temporal changes and the applicability of the findings to contemporary practice.

Lastly, because the study population was restricted to patients who underwent surgery, patients who initiated neoadjuvant treatment but did not subsequently undergo surgery were not captured, potentially leading to an incomplete assessment of treatment patterns, HCRU, and costs.

## 5. Conclusions

This descriptive real-world analysis using linked INHS administrative claims and pathological anatomy database provides insights into treatment patterns, HCRU, direct costs, and clinical outcomes among patients with ER+/HER2– eBC in Italy, with a specific focus on the high-risk subgroup. The findings document a considerable clinical burden and substantial healthcare expenditures during recurrence-free window in patients meeting predefined high-risk criteria, highlighting the importance of risk stratification and the need for effective therapeutic strategies in this population. However, the cost estimates do not encompass expenditures associated with disease recurrence, metastatic progression, or end-of-life care. Further studies incorporating mutually exclusive cohorts and adjusted analyses are needed to better characterise the independent contribution of high-risk clinical features to healthcare costs and outcomes.

## Figures and Tables

**Table 1:** Demographic and clinical characteristics * of patients diagnosed with ER+/HER2– eBC and high-risk ER+/HER2– eBC.

	Cohort #1 Patients with ER+/HER2– eBC	Cohort #2 Patients with High-Risk ER+/HER2– eBC
N. of patients	17,731	366
Demographic characteristics		
Age (years), mean ± SD	62.7 ± 13.1	66.9 ± 14.0
Age (years), median (IQR; min–max)	63 (21; 18–102)	69 (23; 27–94)
18–34 years, *n* (%)	142 (0.8%)	6 (1.6%)
35–64 years, *n* (%)	9389 (53.0%)	147 (40.2%)
≥65 years, *n* (%)	8200 (46.2%)	213 (58.2%)
Clinical characteristics		
CCI, mean ± SD	0.3 ± 0.5	0.4 ± 0.6
CCI: 0, *n* (%)	13,513 (76.2%)	260 (71.0%)
CCI: 1–2, *n* (%)	4144 (23.4%)	101 (27.6%)
CCI: 3–4, *n* (%)	64 (0.4%)	5 (1.4%)
CCI: ≥5, *n* (%)	10 (0.1%)	0 (0.0%)
Comorbidities at baseline		
Cardiovascular comorbidities, *n* (%)	7929 (44.7%)	195 (53.3%)
Diabetes, *n* (%)	1744 (9.8%)	52 (14.2%)
Hyperlipidaemia, *n* (%)	3219 (18.2%)	84 (23.0%)
Liver disease, *n* (%)	204 (1.2%)	4 (1.1%)
Myelodysplastic syndrome, *n* (%)	0 (0.0%)	0 (0.0%)
Neuropathy, *n* (%)	128 (0.7%)	4 (1.1%)
HCV, *n* (%)	25 (0.1%)	0 (0.0%)
HBV, *n* (%)	7 (0.0%)	0 (0.0%)
Therapies at baseline		
Number of drugs prescribed in the 12 months preceding the index-date, mean ± SD	5.5 ± 5.0	6.2 ± 4.8

*Abbreviations*: CCI, Charlson Comorbidity Index; eBC, early breast cancer; ER+, oestrogen receptor-positive; HBV, hepatitis B virus; HCV, hepatitis C virus; HER2–, human epidermal growth factor receptor 2-negative; IQR, interquartile range; SD, standard deviation.

**Table 2:** Treatment patterns in the overall population (Cohort #1) and the high-risk (Cohort #2) patients with ER+/HER2– eBC.

	Cohort #1: Patients with ER+/HER2– eBC	Cohort #2: Patients with High-Risk ER+/HER2– eBC
Surgery, *n* (%)	17,731	366
Lumpectomy/quadrantectomy, *n* (%)	14,295 (80.6%)	196 (53.6%)
Mastectomy, *n* (%)	3436 (19.4%)	170 (46.4%)
Months from first hospitalisation to surgery, mean ± SD	1.6 ± 5.0	2.1 ± 2.9
Duration of hospitalisation (days), mean ± SD	6.4 ± 13.1	5.4 ± 8.0
Neoadjuvant therapy, *n* (%)	709 (4.0%)	40 (10.9%)
CHT, *n* (%) *	699 (98.6%)	40 (100.0%)
RT, *n* (%) *	12 (1.7%)	0 (0.0%)
ET, *n* (%) *	-	-
CHT + ET, *n* (%) *	-	-
Adjuvant therapy, *n* (%)	16,827 (94.9%)	306 (83.6%)
CHT, *n* (%) ^	356 (2.1%)	61 (19.9%)
RT, *n* (%) ^	8825 (52.4%)	157 (51.3%)
ET, *n* (%) ^	13,197 (78.4%)	131 (42.8%)
CHT + ET, *n* (%) ^	3045 (18.1%)	106 (34.6%)
Patients with CHT in both neoadjuvant and adjuvant setting, *n* (%)	183 (1.0%)	14 (3.8%)
Lymph node removal (6 months before index-date), *n* (%)	NI	0 (0.0%)
Lymph node biopsy (6 months before index-date), *n* (%)	5 (0.0%)	0 (0.0%)
Lymph node removal (6 months after index-date), *n* (%)	2229 (12.6%)	133 (36.3%)
Lymph node biopsy (6 months after index-date), *n* (%)	382 (2.2%)	29 (7.9%)

* Percentages calculated on total neoadjuvant treatments for both cohorts; ^ percentages calculated on total adjuvant treatments for both cohorts. *Abbreviations*: CHT, chemotherapy; eBC, early breast cancer; ER+, oestrogen receptor-positive; ET, endocrine therapy; HER2–, human epidermal growth factor receptor 2-negative; IQR, interquartile range; RT, radiotherapy.

**Table 3:** HCRU during the neoadjuvant phase (six months pre-surgery) in total (Cohort #1) and high-risk (Cohort #2) patients with ER+/HER2– eBC.

HCRU During the Neoadjuvant Phase (6 Months Before Surgery)	Cohort #1: Patients with ER+/HER2– eBC (N = 17,731)		Cohort #2: Patients with High-Risk ER+/HER2– eBC (N = 366)	
	Mean ± SD	Median (IQR; Min–Max)	Mean ± SD	Median (IQR; Min–Max)
N. of total claims	9.1 ± 7.5	7 (8; 0–103)	11.7 ± 9.1	9 (10; 0–57)
N. of pharmacy claims	5.0 ± 5.0	4 (6; 0–48)	6.4 ± 5.4	5 (7; 0–27)
Drugs used in neoadjuvant regimen	0.1 ± 0.7	0 (0; 0–16)	0.0 ± 0.4	0 (0; 0–8)
Other *	4.9 ± 4.9	4 (6; 0–48)	6.3 ± 5.4	5 (7; 0–27)
N. of hospitalisation claims	0.1 ± 0.4	0 (0; 0–17)	0.1 ± 0.4	0 (0; 0–3)
Surgery	0.0 ± 0.0	0 (0; 0–0)	0.0 ± 0.0	0 (0; 0–0)
Other *	0.1 ± 0.4	0 (0; 0–17)	0.1 ± 0.4	0 (0; 0–3)
N. of outpatient service claims	4.0 ± 4.3	3 (5; 0–93)	5.2 ± 5.6	4 (4; 0–41)
CHT	0.1 ± 1.3	0 (0; 0–73)	0.4 ± 2.4	0 (0; 0–17)
RT	0.0 ± 0.0	0 (0; 0–2)	0.0 ± 0.0	0 (0; 0–0)
Other *	3.9 ± 3.7	3 (4; 0–79)	4.7 ± 4.1	4 (4; 0–24)

* “Other” refers to non-oncological services or treatments. *Note*: Cohort #2 is a subset of Cohort #1. *Abbreviations*: CHT, chemotherapy; eBC, early breast cancer; ER+, oestrogen receptor-positive; HER2–, human epidermal growth factor receptor 2-negative; IQR, interquartile range; RT, radiotherapy; SD, standard deviation.

**Table 4:** Healthcare direct costs per patient during the neoadjuvant phase (six months pre-surgery) in total (Cohort #1) and high-risk (Cohort #2) patients with ER+/HER2– eBC.

Healthcare Costs (€) ^ per Patient During the Neoadjuvant Phase (6 Months Before Surgery)	Cohort #1: Patients with ER+/HER2– eBC (N = 17,399)		Cohort #2: Patients with High-Risk ER+/HER2– eBC (N = 355)	
	Mean ± SD	Median (IQR; Min–Max)	Mean ± SD	Median (IQR; Min–Max)
Total costs	906.1 ± 1270.4	492 (831; 0–8562)	1377.2 ± 1998.6	644 (936; 0–10,430)
Pharmacy costs	254.9 ± 557.8	90 (251; 0–8028)	379.9 ± 856.7	130 (335; 0–8848)
Drugs used in neoadjuvant regimen	2.1 ± 34.7	0 (0; 0–1987)	3.8 ± 60.7	0 (0; 0–1122)
Other *	252.8 ± 552.4	90 (250; 0–8028)	376.1 ± 849.8	130 (335; 0–8848)
Hospitalisation costs	169.2 ± 766.0	0 (0; 0–8306)	268.2 ± 1019.3	0 (0; 0–6586)
Surgery	0.0 ± 0.0	0 (0; 0–0)	0.0 ± 0.0	0 (0; 0–0)
Other *	169.2 ± 766.0	0 (0; 0–8306)	268.2 ± 1019.3	0 (0; 0–6586)
Outpatient services costs	482.0 ± 754.9	255 (537; 0–8236)	729.0 ± 1284.6	373 (604; 0–10,011)
CHT	27.0 ± 359.4	0 (0; 0–6820)	166.0 ± 924.5	0 (0; 0–7463)
RT	1.9 ± 85.3	0 (0; 0–4809)	0.0 ± 0.0	0 (0; 0–0)
Other *	453.1 ± 630.9	254 (533; 0–8236)	563.1 ± 664.2	371 (582; 0–4502)

^ Costs are inflation-adjusted to 2024 Euros (healthcare Consumer Price Index—CPI); deaths and cost outliers were excluded. * “Other” refers to non-oncological services or treatments. *Note*: Cohort #2 is a subset of Cohort #1. *Abbreviations*: CHT, chemotherapy; eBC, early breast cancer; ER+, oestrogen receptor-positive; HER2–, human epidermal growth factor receptor 2-negative; IQR, interquartile range; RT, radiation therapy; SD, standard deviation.

**Table 5:** Annualised HCRU per patient during follow-up in total (Cohort #1) and high-risk (Cohort #2) patients with ER+/HER2– eBC (deaths excluded).

Annualised HCRU per Patient During Follow-Up	Cohort #1: Patients with ER+/HER2– eBC (N = 16,971)		Cohort #2: Patients with High-Risk ER+/HER2– eBC (N = 297)	
	Mean ± SD	Median (IQR; Min–Max)	Mean ± SD	Median (IQR; Min–Max)
N. of total claims	26.3 ± 12.5	24.2 (14.4; 0.3–193.3)	30.3 ± 14.2	27.8 (16.5; 4.9–90.8)
N. of pharmacy claims	15.4 ± 8.8	13.7 (10.1; 0.0–117.2)	17.3 ± 10.0	15.1 (11.7; 0.6–57.3)
Drugs used in adjuvant regimen	5.6 ± 2.8	5.7 (2.9; 0.0–26.6)	4.9 ± 3.3	5.5 (4.9; 0.0–13.0)
Other *	9.8 ± 8.2	7.8 (9.4; 0.0–109.5)	12.5 ± 9.8	10.1 (11.1; 0.5–57.3)
N. of hospitalisation claims	0.6 ± 0.7	0.4 (0.5; 0.1–26.7)	0.9 ± 0.9	0.7 (0.8; 0.1–5.0)
Surgery	0.3 ± 0.3	0.2 (0.3; 0.1–4.1)	0.5 ± 0.4	0.3 (0.3; 0.1–3.0)
Other *	0.3 ± 0.6	0.1 (0.4; 0.0–23.7)	0.5 ± 0.6	0.3 (0.7; 0.0–3.9)
N. of outpatient services claims	10.3 ± 6.9	9.1 (7.4; 0.0–156.5)	12.0 ± 7.5	10.3 (8.6; 0.0–43.7)
CHT	0.2 ± 1.3	0.0 (0.0; 0.0–127.5)	0.5 ± 1.5	0.0 (0.0; 0.0–12.2)
RT	0.7 ± 1.8	0.0 (0.6; 0.0–39.9)	0.7 ± 1.6	0.0 (0.6; 0.0–13.2)
Other *	9.5 ± 6.0	8.4 (6.6; 0.0–148.5)	10.8 ± 6.4	9.3 (7.0; 0.0–39.3)

*Note*: Patients with ≥6 months of follow-up were included (N = 16,971) to avoid bias from annualising short observation periods. * “Other” refers to non-oncological services or treatments. *Abbreviations*: CHT, chemotherapy; eBC, early breast cancer; ER+, oestrogen receptor-positive; ET, endocrine therapy; HCRU, healthcare resource utilisation; HER2–, human epidermal growth factor receptor 2-negative; IQR, interquartile range; RT, radiotherapy; SD, standard deviation.

**Table 6:** Annual healthcare direct costs per patient during follow-up in total (Cohort #1) and high-risk (Cohort #2) patients with ER+/HER2– eBC.

Annual Healthcare Costs ^ (€) per Patient During Follow-Up	Cohort #1: Patients with ER+/HER2– eBC (N = 16,696)		Cohort #2: Patients with High-Risk ER+/HER2– eBC (N = 290)	
	Mean ± SD	Median (IQR; Min–Max)	Mean ± SD	Median (IQR; Min–Max)
Total costs	4671.4 ± 3018.9	3847.1 (3100.1; 38.9–19,497.9)	6934.0 ± 5185.3	5139.6 (4675.9; 1145.3–27,975.9)
Pharmacy costs	1374.2 ± 1253.4	1099.5 (1048.2; 0.0–17,668.1)	1512.7 ± 1978.8	1149.5 (900.2; 34.3–18,724.0)
Drugs used in adjuvant regimen	509.2 ± 499.9	498.2 (588.9; 0.0–13,844.0)	589.7 ± 758.9	575.4 (726.2; 0.0–6412.2)
Other *	865.0 ± 1118.4	505.6 (911.4; 0.0–17,116.9)	923.0 ± 1773.7	524.1 (772.6; 8.6–18,679.6)
Hospitalisation costs	1838.4 ± 1973.1	1229.3 (1588.8; 0.0–18,477.2)	3658.1 ± 4009.0	2331.7 (3121.5; 272.4–25,432.4)
Surgery	987.5 ± 913.6	723.1 (840.9; 0.0–12,907.7)	1547.7 ± 1344.8	1175.1 (1070.2; 186.1–9076.8)
Other *	850.9 ± 1636.9	26.5 (1019.3; 0.0–17,068.7)	2110.4 ± 3532.6	789.1 (2714.6; 0.0–21,284.6)
Outpatient services costs	1458.8 ± 1543.4	1013.0 (1345.6; 0.0–16,181.3)	1763.2 ± 1653.2	1251.2 (1747.8; 0.0–9653.7)
CHT	27.5 ± 199.5	0.0 (0.0; 0.0–5337.7)	147.6 ± 499.6	0.0 (0.0; 0.0–4013.0)
RT	692.9 ± 1294.3	0.0 (845.5; 0.0–15,827.5)	614.5 ± 1043.5	0.0 (773.8; 0.0–6605.9)
Other *	738.4 ± 644.9	570.3 (580.4; 0.0–9754.4)	1001.1 ± 784.9	774.8 (904.3; 0.0–5075.2)

^ Costs are inflation-adjusted to 2024 Euros (healthcare CPI); deaths and cost outliers were excluded. * “Other” refers to non-oncological services or treatments. *Abbreviations*: CHT, chemotherapy; eBC, early breast cancer; ER+, oestrogen receptor-positive; ET, endocrine therapy; HER2–, human epidermal growth factor receptor 2-negative; IQR, interquartile range; RT, radiotherapy; SD, standard deviation.

## Data Availability

In accordance with local laws and regulations, the data analysed are not publicly available.

## Abbreviations

AI	Aromatase inhibitor
ATC	Anatomical Therapeutic Chemical
BC	Breast cancer
CCI	Charlson Comorbidity Index
CDK4/6i	Cyclin-dependent kinase 4 and 6 inhibitor
CHT	Chemotherapy
DRG	Diagnosis-Related Group
eBC	Early breast cancer
ER+	Oestrogen receptor-positive
ET	Endocrine therapy
HBV	Hepatitis B virus
HCRU	Healthcare resource utilisation
HCV	Hepatitis C virus
HER2–	Human epidermal growth factor receptor 2-negative
ICD-9-CM	International Classification of Diseases, Ninth Revision, Clinical Modification
INHS	Italian National Health Service
IQR	Interquartile range
LHU	Local Health Unit
M0	No distant metastasis
NI	Not issuable
rwOS	Real-world overall survival
RT	Radiotherapy
rwEFS	Real-world event-free survival
SD	Standard deviation
